# Separating the Wheat from the Chaff: Nutritional Value of Plant Proteins and Their Potential Contribution to Human Health

**DOI:** 10.3390/nu12082410

**Published:** 2020-08-12

**Authors:** Robert W. Davies, Philip M. Jakeman

**Affiliations:** 1 Physical Education and Sport Sciences Department, University of Limerick, V94 T9PX Limerick, Ireland; phil.jakeman@ul.ie; 2 Health Research Institute, University of Limerick, V94 T9PX Limerick, Ireland

**Keywords:** amino acids, climate change, dietary proteins, essential amino acids, humans, lysine, plant proteins, protein quality, recommended dietary allowances, sustainable development

## Abstract

The quality and nutritional value of dietary proteins are determined by the quantity, digestibility and bioavailability of essential amino acids (EAA), which play a critical role in human growth, longevity and metabolic health. Plant-source protein is often deficient in one or more EAAs (e.g., branched-chain amino acids, lysine, methionine and/or tryptophan) and, in its natural form, is less digestible than animal-source protein. Nevertheless, dietary intake of plant-source protein has been promoted because of its potential health benefits, lower cost of production and lower environmental impact compared to animal-source protein. Implementation of dietary strategies that improve both human and planetary health are of critical importance and subject to growing interest from researchers and consumers. Therefore, in this review we analyse current plant protein intake patterns and discuss possible countermeasures that can enhance plant protein nutrition, examples include: (1) combining different plant proteins with complementary EAA profiles; (2) identification and commercial cultivation of new and novel high-quality plant proteins; (3) industrial and domestic processing methods; and (4) genome-editing techniques.

## 1. Introduction

Dietary protein is an important macronutrient that plays a critical role in human health and longevity [1]. All 20 amino acids (AA) are required, in adequate amounts, for tissue protein synthesis and maintenance of normal metabolic function [1,2,3,4]. The nine essential amino acids (EAA) are particularly important as they cannot be synthesised in human cells at sufficient rates to meet metabolic demand (MD), so must be obtained from dietary protein sources [1,2,3,4]. Recommended dietary allowance (RDA) for protein for adults is currently ~0.8 g·kg^−1^·d^−1^, but is higher for populations with greater MD (e.g., infants, children, pregnant/lactating women, athletes) [5]. In developing regions, protein malnutrition is a major issue [6,7], whereas in developed regions protein intake is generally sufficient and discussions are based on whether higher protein intakes are positively or negatively impact health and fitness outcomes [1,8]. 

Over the last 60 years, the per capita global protein consumption has increased [9], but rapid population growth and climate change threaten food security, and human and planetary health [6,10,11,12,13,14]. In response, scientists and politicians have encouraged a transition away from animal- to plant-sourced foods as a means to meet sustainable development goals (SDGs) [11,12,13,14]. However, the affordability, validity and nutritional adequacy of plant-based diets have been questioned [15,16,17].

Plant crops are the primary harvesters of solar energy acting as a cost and energy-efficient nutrient-rich sink—and a valuable source of protein, carbohydrate, essential vitamins and minerals [18]. Hence, by necessity or choice, plant foods and plant-based diets have been popular throughout human history, with large segments of the population adopting them because of personal, ethical, religious or philosophical beliefs. Consequently, we deem it pertinent to evaluate and review dietary strategies that can be used to enhance the nutritional value of plant-sourced protein (PSP) for human consumption.

## 2. Plant Protein Consumption: Trends and Patterns

**Figure 1:**
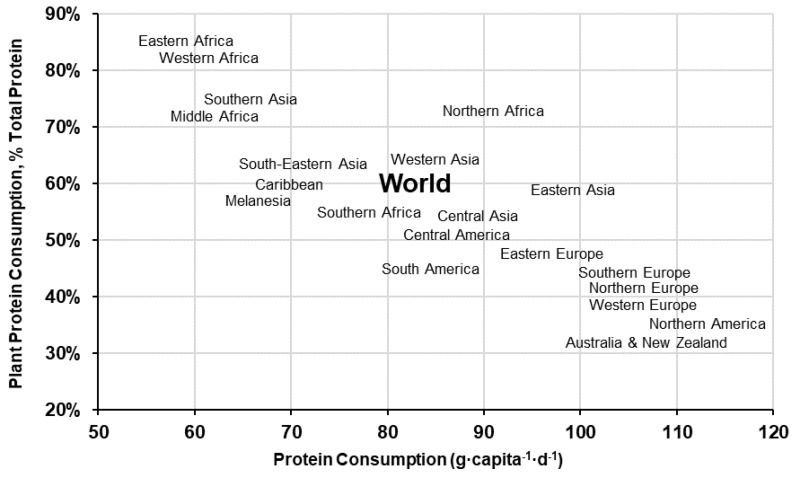
World and regional percent plant protein consumption and total daily protein consumption (g·capita^−1^·d^−1^) [9].

**Figure 2:**
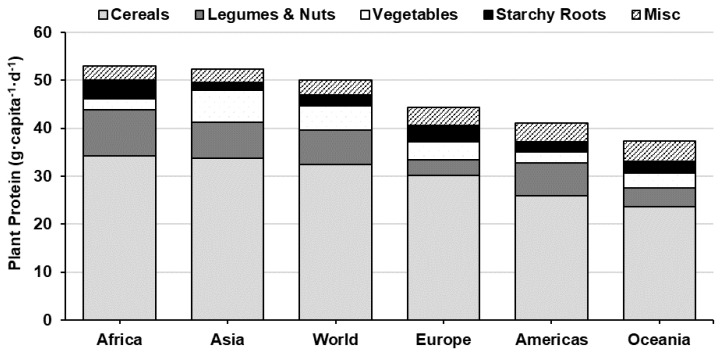
World and continental plant protein consumption by source (g·capita^−1^·d^−1^) [9]. Misc. includes protein from fruit, seeds, spices, stimulants and aquatic plants.

Hundreds of plant species have been identified and commercially cultivated for human consumption, however, most of the world’s population only depend on ~20 plant crops-grouped into a few broad categories (e.g. cereals, legumes, vegetables, starchy roots, fruit and nuts) [9,18]. Supply data from the Food and Agriculture Organisation Corporate Statistical Database (FAOSTAT) indicate that PSP accounts for ~60% of the global protein supply (50 g·capita^−1^·d^−1^ plant protein of 83 g·capita^−1^·d^−1^ total protein) [9], but varies significantly by region—ranging from over 80% in sub-Saharan Africa to under 40% in Western Europe, North America and Oceania (Figure 1). Historical data recorded over the last 60 years show that global protein consumption has increased 18 g·capita^−1^·d^−1^ (64 g·capita^−1^·d^−1^ in 1967 to 82 g·capita^−1^·d^−1^ in 2017) and the percent contribution of animal-sourced protein (ASP) by 9 percentage points [9]—driven by a rapid increase in ASP consumption in developing regions [19]. Globally, cereal crops are the most widely consumed PSP across all regions, where three cereal crops (i.e., wheat, rice and maize) account for 36% of all protein consumed and 60% of the PSPs [9] (Figure 2).

## 3. Human Protein and Amino Acid Requirements

In humans, the nutritional value of dietary protein is determined by its ability to meet protein and AA requirements for growth, maintenance and basic physiological needs [1,2,3,4,5]. However, over the last century the mensuration of human physiological needs and, consequently, protein/AA requirements have been widely debated [2,3,4,5]. Dietary protein/AA provide nitrogen (N), which is required to support basic metabolic processes such as synthesising new tissue proteins and producing nitrogenous compounds that support metabolism (e.g. hormones, neurotransmitters, creatine phosphate, purines and pyrimidine bases). Therefore, in theory, protein/AA requirements are directly proportional to MD [2,3]. Specifically, the supply of the nine EAAs (i.e., histidine (His), isoleucine (Ile), leucine (Leu), lysine (Lys), methionine (Met), phenylalanine (Phe), threonine (Thr) and valine (Val)), and conditionally essential AA under certain conditions (e.g., arginine (Arg), asparagine (Asp), cysteine (Cys), tyrosine (Tyr), taurine (Tau), glycine (Gly), glutamine (Glu) and proline (Pro)) are required as they cannot be biosynthesised at an adequate rate to meet MD [2,3,4,18].

For adolescents and adults, a RDA of ~0.8 g·kg^−1^·d^−1^ (~132 mg N·kg^−1^·d^−1^) is recommended for N-balance (i.e., N-loss equals N-intake), with the estimated average requirement (EAR) of protein (i.e., minimum N intake required to attain N-balance for half of the population) set at 0.66 g·kg^−1^·d^−1^ [5,20]. But, those with higher MD (e.g., children, elderly, pregnant/lactating women, athletes and some clinical populations) generally require greater amounts of protein (>1.0 g·kg^−1^·d^−1^) [5,20,21,22]. It is important to note that RDA estimates derived from N-balance data represent the most efficient use of dietary protein (i.e., the amount needed to offset protein losses through metabolism and excretion) [3,21,22]. However, these recommendations fail to consider that several desirable physiological outcomes occur at intakes > RDA values, which reflect a minimal rather than an optimal level of protein intake [4,21,22,23]. Indeedmore recently, higher RDA estimates (i.e., >1.0 g·kg^−1^·d^−1^) have been (re)calculated and (re)quantified via segmented regression and the indicator amino acid oxidation (IAAO) technique [21,22].

## 4. Protein Quality

In addition to the protein RDA, the quality of the protein must be considered as it is inextricably linked to RDA and protein adequacy. Protein quality is based on the ability of the protein to deliver the individual EAAs in sufficient amounts to meet requirements. Indeed, several studies have reported differences between isonitrogenous PSP and ASP, showing that PSP is utilised with a lower efficiency, implying that PSP is lower-quality than ASP [20]. 

Quantitative assessment of dietary protein quality was devised to reflect postprandial bioavailability of the EAAs in relation to requirements, determined by the content and profile of EAA and the true ileal digestibility of the protein. Although several methods exist to evaluate protein quality, the digestible indispensable amino acid score (DIAAS) has been adopted by the World Health Organisation (WHO)/Food and Agriculture Organisation (FAO)/United Nations University (UNU) [24,25]. The DIAAS is calculated as the mg amount of digestible EAA in 1 g of dietary protein (mixed or single-source) divided by the mg amount of the digestible EAA in 1 g in an ‘ideal’ reference protein [24,25]. For foods susceptible to damage from processing, digestible reactive (bioavailable) Lys is used rather than total digestible Lys [24]. The lowest scoring EAA is the DIAAS and the first limiting EAA (LEAA). Consequently, for the EAR of protein (0.66 g·kg^−1^·d^−1^) the DIAAS represent the fraction of the minimum daily requirement of the first LEAA consumed. Thus, 0.66 g·kg^−1^ of protein with a DIAAS of 1.0 will, in theory, supply 100% of the minimum daily requirements of the first LEAA and all other EAAs [24,25]. If the protein has a DIAAS of >1.0 the protein is considered ‘excellent/high’ quality, >0.75 ‘good’ quality and <0.75 no protein quality claim can be made. Generally, ASP is considered ‘excellent’ quality, whilst PSP, in its natural form, is generally considered ‘low’ quality with most scoring <0.75 (Table 1).

In the main cereal crops, Lys is the first LEAA, and the sulphur-containing amino acids (SAA: Met and Cys) are usually the first LEAA in the legume crops, although there are exceptions where Trp (e.g., haricot beans, split yellow peas, chickpea, pinto beans) or Lys (black-eyed peas) is the first LEAA (Table 1), but it should be noted that PSPs also have lower branched-chain amino acid (BCAA) (Ile, Leu, Val) and Trp scores than most ASPs (Table 1) [7,18]. In the last decade, some new and novel high-quality PSPs have been identified and researched (e.g., fungi, pseudo-cereals, aquatic plants and algae), however, they are yet to be commercially cultivated for human consumption [26]. 

Protein RDA is based on high-quality protein intake (i.e., DIAAS > 1.0) [24,25], but for lower-quality proteins (i.e., DIAAS < 0.75) a larger quantity of protein would have to be consumed to meet protein/AA requirements. For example, if dietary protein consumption was entirely dependent on three main cereal staples (i.e., wheat, rice and maize), the DIAAS (based on a weighted mean average (Table 1)) would be ~0.5 (LEAA: Lys). Consequently, to meet minimum requirements for Lys, the EAR of protein would need to double (i.e., >1.3 g·kg^−1^·d^−1^). However, in most instances fold increases in PSP intake would not be necessary to meet requirements as normally multiple different dietary PSPs and ASP are consumed [9]. Nevertheless, it is necessary to consider protein quality alongside RDA, particularly for populations with higher MD and thus, EAA and protein requirements (e.g., infants, children, pregnant, elderly, physically active, athletes) acute and/or chronic disease (e.g., pneumonia, gastrointestinal infection, cachexia/sarcopenia) limited access to a variety of PSPs and/or consuming insufficient quantities of food and protein in general [5,6,7,20,21,22]. 

As the DIAAS is calculated per gram of protein, comparative evaluation of the DIAAS between different protein sources is conducted on an isonitrogenous basis [24]. Consequently, the DIAAS fails to consider that, in their natural form, plant-sourced foods typically have a lower protein density (i.e., unit of protein mass per unit of product mass) and higher caloric density (i.e., kcal per unit of protein mass) compared to animal-source foods [4]. For example, to provide a 0.3 g·kg^−1^ of protein (i.e., a meal-sized serving), the caloric equivalent for soybeans would be higher than poultry meat despite having similar DIAAS (3.4 kcal·kg^−1^ vs. 1.9 kcal·kg^−1^) [27]. Animal-source foods with low protein density (e.g., whole milk at 3–4% protein) would also have higher caloric equivalents (4.9 kcal·kg^−1^), but score markedly better than low-quality, low-density plant foods (e.g., 14.6 kcal·kg^−1^ for wheat flour and 13.9 kcal·kg^−1^ for groundnuts). In most instances, consuming greater amounts of PSP to compensate for inferior protein quality is not always feasible and may negatively impact health (e.g., obesity, metabolic syndrome, malnutrition and gastrointestinal distress due to overconsumption of calories, carbohydrate, dietary fibre and antinutrients). Therefore, it is pertinent that we review dietary strategies that enhance the quality of PSP [28,29,30,31]. 

## 5. Protein Complementation

As the DIAAS is expressed in standardised units (i.e., mg of EAA per g of protein), different protein sources can be compounded at discrete time intervals (e.g., per meal or per diem basis). In this approach, two complementary protein sources (with different LEAAs) can be combined to affect the DIAAS, greater than either individual protein. For example, 100 g serving of rice (Oryza sativa) and 100g of lentils (Lens culinaris) would be classified, individually, as low-quality protein sources (DIAAS of 0.74 and 0.54, respectively) [33]. Whereas a 100 g serving of 65 g of rice and 35 g of lentils has a high-quality score (DIAAS = 1.0), because rice and lentils have complementary EAAs (i.e., rice is deficient in Lys (DIAAS = 0.54) but has sufficient amounts of the SAAs (DIAAS = 2.05), whereas lentils are SAA deficient (DIAAS = 0.54) but Lys sufficient (DIAAS = 1.53)), which together form a ‘balanced’ EAA profile [24].

Cereal and legume crops are generally complementary (Table 1). However, current global consumption patterns indicate that cereal and legume proteins are consumed at an imbalanced ratio of 6:1 (i.e., 6 g of cereal protein is consumed per 1 g of legume protein)—where a ~2:1 ratio would be required to optimise PSP quality scores [9,24]. In developed regions, imbalanced intake of PSP is not a major issue as is typically offset by ASP consumption, which accounts for ~40% global protein consumption (regional range: 16% in East Africa to 65% in Oceania) [9,10,18,24]. Nevertheless, if ASP intake is low or to be lowered, attention needs to be directed towards the quality of the PSP component of the diet. As such, protein complementation is a well-known, long-established, but often overlooked dietary strategy to improve PSP quality.

## 6. Timing and Distribution

The RDA for protein is normally expressed per diem, which does not consider the timing (i.e. the time between eating occasion (EO)) or distribution (i.e. the number of EO and amount of protein per EO) of protein consumption throughout the day. For PSP, it should be noted that the LEAA Lys (and Thr) are unique among the EAAs as it appears that they can be conserved, indicating that they do not need to be co-ingested with the other EAAs at each EO (i.e., fed ‘out-of-phase’) [2]. Evidence for this comes from the analysis of the muscle tissue EAA pool 7 h after ingestion of albumin protein, which reports Lys (and Thr) concentrations twice that of the other EAAs (also remaining above baseline-levels) [39]. The conservation of Lys (and Thr) is thought to be related to its slower turnover rate, more efficient recycling from tissue protein and larger free-pools of Lys compared to other EAAs (e.g., BCAA and Trp) [2,39]. Thus, it is possible that the endogenous pool of Lys could be utilised to meet postprandial MD if the dietary protein source is deficient in Lys [2,18,39].

In addition to providing nitrogen, some other AAs also activate signalling pathways that regulate metabolism (e.g., protein turnover, immune function, mitochondrial activity, fatigue, satiety, cognitive function, and lipid and glucose metabolism) [1,2,4,23]. Each signalling pathway is sensitive to transient changes in postprandial plasma and intracellular AA concentrations, and thus, dependent on the temporal pattern of protein intake throughout the day. The effect that the timing and distribution of high-quality ASP has been robustly investigated over the last two decades—and there is some consensus on the optimal dose per EO (i.e., >0.3 g·kg^−1^) and distribution of protein throughout the day (i.e. equally balanced across 3–5 EOs consumed at 3–5 h intervals) for muscle protein synthesis—yielding per diem equivalents higher than the current RDA (i.e., >0.9 g·kg^−1^·d^−1^) [22,23,40,41]. 

In theory, postprandial deficiencies in Lys (and Thr) in PSP can be drawn from body-pools, but postprandial deficiencies in other EAAs (e.g., BCAA, particularly Leu, and Trp) [2], would limit postprandial anabolic/metabolic potential. For example, to reach an equivalent quantity of digestible Leu from a milk protein (2.0 g Leu per 20 g protein), higher doses of wheat protein (1.1 g Leu per 20 g protein) or soy protein (1.4 g Leu per 20 g protein) would need to be consumed (i.e., 36 g and 29 g respectively) [24,27,34]. In a recent study comparing postprandial myofibrillar protein synthesis (myoPS) in healthy older adults, 35 g of casein protein stimulated an increase in myoPS, whereas, a 35 g of wheat protein did not [42]. However, a 65 g dose of wheat protein (delivering the same amount of Leu as 35 g of casein) robustly stimulated myoPS [42], and in a separate study 32 g of fungal protein (Fusarium venenatum) stimulated postprandial myoPS to a greater extent than a Leu-matched 26 g bolus of milk protein [43].

If available and consumed in appropriate amounts and/or combinations, it appears that adequate levels of protein/AA nutrition can be attained from PSP. However, whether optimal levels of protein/AA nutrition can be achieved exclusively from PSP, especially for clinical and athletic populations with higher MDs, is questionable—as, in its natural form, higher intakes from PSP may not be practicable and may negatively affect health (e.g., obesity, metabolic syndrome, malnutrition and gastrointestinal distress due to overconsumption of calories, carbohydrate, dietary fibre and antinutrients). 

## 7. Digestibility and Bioavailability

In addition to the content and profile of EAA, the quality and the nutritional value of the protein is affected by the true ileal digestibility of the protein. PSP, in its natural form, generally has lower digestibility than ASP (75–80% *vs.* 90–95%) (Table 1), due to its stronger cell walls, seed-coats and microstructural arrangement of the protein (e.g., protein cross-linking, secondary beta-conformation and protein chain rigidity), antinutritional factors (ANFs) (e.g., protease inhibitors, lectins, polyphenols, tannins, phytic acid) and a high non-protein component (NPC) (e.g., insoluble dietary fibre and non-starch polysaccharides) [44,45,46]. However, prior to human consumption most plant foods undergo some form of domestic or commercial processing—usually performed to increase shelf-life, palatability and/or change some other functional properties of the food (e.g., solubility, binding-capacity, texture, colour) rather than to increase nutritional value [46]. Nevertheless, processing methods can be also used to improve the digestibility, and thus, the quality and nutritional value of PSP [45,46].

Indeed, there are several domestic processing methods that have been shown to improve PSP digestibility (e.g., heating, dehulling, soaking, autoclaving, microwaving, drying, germination and fermentation) [46]. For example, boiling peas (*Pisum sativum* L.) in water increases protein digestibility (75% up to 80%) [47]. Industrial processing methods (e.g., milling, microfiltration, alkali-solubilisation acid precipitation, centrifugation, flocculation, fractionation, enzymatic-hydrolysis, ultra-heat treatment and spray-drying) can increase protein digestibility of peas, and other plants, up to 97% (Table 1). Improved digestibility of PSP through domestic and/or industrial processing is a result of inactivating ANFs and changing the structural and functional properties of the plant—whereby partial denaturation of the tertiary and quaternary structure makes the PSP more susceptible/accessible to the digestive proteases [45,46]. However, it should be noted that some thermal and chemical processes may also reduce digestibility and nutritional value [45,46]. In particular, non-enzymatic browning as a consequence of the Maillard reaction (used in roasting, baking or frying), appears to negatively affect protein conformation and digestibility by increasing protein crosslinking, protein aggregation, protein–protein interaction and protein glycation [46,48]. For example, during baking, the wheat protein in the bread crumb and crust are exposed to different heat intensities (~100 °C and ~180 °C, respectively) leading to different digestibility scores, with the crust having lower digestibility than the crumb [48].

The ease by which the peptides and AA can be liberated from the microstructure influences digestibility and nutritional value. For PSP, complete elimination of the microstructure and the NPC appears to enhance digestibility and protein quality [37,46,47]. Still, the role of the food structure, nutritional value of the NPC and the interaction between the protein and NPC must be considered (collectively termed the food matrix effect) [49]. The food matrix effect can be synergistic or antagonistic, defined by the desired outcome of the diet. For example, dietary fibre and the ANFs contained in plant food generally reduces digestibility and protein quality [44,45]. However, dietary fibre is considered beneficial for other aspects of health (e.g., gastrointestinal function, immune function, lipid and glucose metabolism) [45,46]. Plant ANFs such as polyphenols and phytic acid act in a similar manner having antioxidant, anti-inflammatory, antibacterial effects [45]. Whilst domestic processing methods have been reasonably well investigated, there is a paucity of research focused upon commercial processing methods specifically designed to enhance PSP quality [46]. Hence, the degree, accuracy and efficiency that commercial processing methods can be used to enhance PSP quality remains an ongoing scientific and technological challenge.

## 8. Genetic Engineering and Selective Breeding

PSP is usually deficient in one or more EAAs (i.e., BCAAs, Met, Lys, Trp) (Table 1) as they lack the enzymes for de novo biosynthesis. The three main cereal crops (i.e. wheat, rice and maize) are deficient in Lys and heavily relied upon in developing regions [9]. Hence, over the last 50 years, several approaches have been developed to improve the Lys content of cereal crops. An early indication that the genome could be altered to improve AA-content of plants was the discovery of the opaque-2 mutation, which increased the Lys and Trp content of maize [50]. After its discovery, the opaque-2 gene was used normal inbred lines with mixed results—effectively increasing Lys but also causing abnormal phenotypic traits and lower-crop yields [51]. In the 1990s the opaque-2 mutant was used in a parent line to develop quality protein maize (QPM), which had comparable phenotypic traits and crop-yield to other maize cultivars, but with a two-fold increase in the content and greater bioavailability of Lys (~90%) [30,51]. In populations that are reliant on maize, a substitution of maize for QPM was shown to increase growth weight and height in infants and children with mild to moderate undernutrition [30]. Despite the commercial and nutritional importance of the QPM, attempts to breed for similar genotypes in other cereals crops has been unsuccessful, calling for alternative methods [51]. 

There are inherent difficulties in classical genetic and breeding approaches used to increase the nutritional value PSP, due to the tightly regulated Lys metabolic pathway and their deleterious effects crop yields and plant growth [51]. Thus, other genetic engineering approaches have been used to good effect. To date, only two genetically modified (GM) events have been commercialised in cereal crops to modify AA-traits [52]. These are the dapA-gene (Corynebacterium glutamicum), which increases free-Lys content and the cor-dapA gene, which encodes the enzyme that catalyses the first reaction in the Lys biosynthetic pathway [52].

There is the potential that GM plants can significantly improve PSP quality and nutritional value, however, the biosafety and bioefficacy have yet to be confirmed in humans. Moreover, even though GM crops are only granted regulatory approval after strict assessment (i.e., toxicity, allergenicity, compositional analysis, ecological impact), like all other GM crops, the opportunity to improve PSP quality via GM will be conditional on public acceptance [51,52]. To address some of the major concerns with transgenic GM crops, alternative techniques have been developed (e.g., cisgenesis, intragenesis and genome editing (GEd)) that do not require foreign genes. Indeed, GEd has been used in plant crops to target the gene that encodes the enzyme acetolactate synthase (ALS), which catalyses the first step in the biosynthesis pathway of the BCAAs [53,54]. To improve PSP quality, further efforts using GEd technology can be used to increase biosynthesis, block catabolism, and increase expression of recombinant proteins enriched in EAA in plants [51]. Given the high-precision of GEd systems such as transcription activator-like effector nucleases (TALENs) and the clustered regularly interspaced short palindromic repeats (CRISPR)/Cas9) system, development of higher-quality PSP can be expected in future. Furthermore, since the GEd plants would be indistinguishable from crops developed via selective breeding, there is hope that cultivation and commercialisation of GEd crops will be more rapidly and broadly accepted than transgenic crops.

## 9. Sustainability and Nutrition

**Figure 3:**
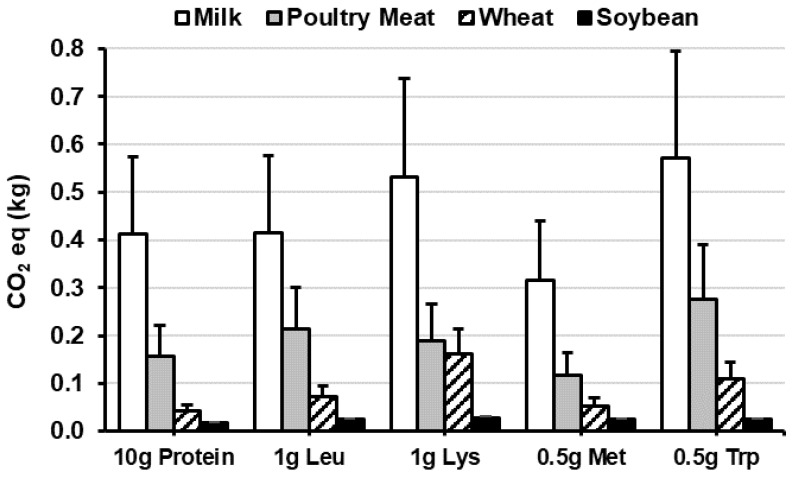
Carbon dioxide (CO_2_) emission equivalents per g dose of digestible protein and the essential amino acids (Leu, Lys, Met, Trp) for whole milk, cooked poultry meat, wheat and soybean flour. Data are mean + standard deviation (SD). CO_2_ data [57]; nutrient data [24,27].

The 2019 special report by the Intergovernmental Panel on Climate Change (IPCC) describes plant-based diets as a ‘major opportunity for mitigating and adapting to climate change’, which includes a policy recommendation to reduce meat consumption [13]. Consequently, great emphasis has been placed on plant foods and plant-based diets to align with the SDGs and reduce the environmental footprint associated with food production (e.g., greenhouse gas emissions (GHGE), climate change, land-use, energy-use and water-use) [11,12,13,14]. However, several criticisms have been raised, related to the affordability and validity of plant-based diets/dietary recommendations [15,16,17], and in particular, the reliance on modelling studies that make favourable and simplistic assumptions about dietary substitutions and nutritional adequacy [16]. For instance, the environmental impact of different protein sources is often compared per unit of mass, per serving, per kcal or protein mass [11,12], but when the nutritional value and quality of the protein is considered the differences between some ASPs and PSPs are negligible [55,56] (Figure 3). For example, recalculating GHGE equivalents [57] for the two most commonly consumed ASPs (milk and poultry meat [9]) against wheat and soy (most commonly consumed cereal and legume crops) indicates that poultry meat and wheat have similar GHGE equivalents for Lys (0.19 kg CO_2_·g Lys^−1^ *vs.* 0.16 kg CO_2_·g Lys^−1^), and larger disparities exist between different PSPs (i.e., wheat vs. soybeans) and ASPs (i.e., milk vs. poultry meat), than between ASP and PSP (e.g. wheat vs. poultry meat) (Figure 3).

SDGs aim to meet the needs of the present, without compromising the ability of future generations to meet their own needs [58]. Animal-source foods provide EAAs and other essential nutrients that are difficult to obtain in sufficient quantities exclusively from plant-source foods (e.g., vitamin B_2_, B_12_, D_3_, heme-iron, iodine, calcium, essential fatty acids and zinc). Thus, from a nutritional perspective, there is justification for modest increases in animal food intake in developing regions, particularly those with higher MD [17]. Additionally, there is ample room to curtail inefficiencies in animal food production (e.g., farming methods, processing, packaging and distribution) and consumption patterns (e.g., food waste and overconsumption) in developed regions. However, if for ideological or political reasons, a transition away from animal to plant-sourced foods is (en)forced, then improving the quality and nutritional value of PSP, and the methods used to assess protein quality and dietary adequacy, is of critical importance for the health of both current and future generations.

## 10. Summary and Conclusions

In this brief review, the nutritional value of PSP related to human protein/AA requirements is highlighted and discussed. Increasing the RDA >1.0 g·kg^−1^·d^−1^ may be a simple and effective strategy to offset protein/AA deficiencies in PSP, however, in practice, this strategy may not always be practicable or efficacious—particularly for those who rely on a limited variety of PSPs by necessity, not by choice. In developed regions, increasing intake of PSP should be little concern as dietary strategies such as protein complementation, supplementation and fortification of PSP with EAA can be implemented to help meet protein/AA requirements. This is fortunate as increased pressure to find sustainable food sources has led to a trend away from animal- to plant-source foods and plant-based diets. At present, it appears that there is ample room to improve sustainable intensification of animal-source food production, whilst simultaneously increasing in the intrinsic nutritional value of plant-source foods via GEd or processing methods. This dual approach will favourably impact global nutrition and the health of both current and future generations, but remains a significant scientific and technological challenge.

## Figures and Tables

**Table 1:** Global plant protein supply (% total plant protein intake), digestibility and digestible indispensable amino acid score (DIAAS) range. CP, crude protein; whey protein concentrate (WPC) and whole milk powder (WMP) are provided as animal protein references. Leu, leucine; Lys, lysine; SAA, sulphur amino acids (methionine and cysteine); Trp, tryptophan. First limiting amino acid is in bold. ^a^ World Health Organisation (WHO)/Food and Agriculture Organisation (FAO)/United Nations University (UNU) adult indispensable amino acid (IAA) reference pattern as mg IAA·g protein^−1^ (Leu, 66; Lys, 57; SAA, 27; Trp, 8.5) [24].

Plant Source	Global Supply %	Protein % Mass	CP True Ileal Digestibility %	DIAAS ^a^			
				Leu	Lys	SAA	Trp
Wheat [24,27,32,33,34,35,36]	32	11–17	71–94	0.82–1.06	**0.20–0.54**	0.64–1.51	1.15–1.62
Rice [27,32,33,35,36,37]	21	8–9	73–90	0.84–1.17	**0.37–0.73**	0.40–2.11	0.84–2.29
Maize [27,32,36,37]	8	7–9	70–76	1.31–2.01	**0.48–0.54**	0.68–1.46	0.70–1.04
Pulses [27,33,34,36,37,38]	5	22–30	78–90	0.97–1.16	1.05–1.53	**0.46–0.85**	0.78–1.82
Beans [27,36,37,38]	3	23–25	58–83	0.72–1.09	0.93–0.98	**0.49–0.60**	0.76–1.86
Potatoes [36,27]	3	2–3	52–58	0.34–0.39	0.42–0.46	0.38–0.77	0.49–1.42
Soya [27,34,36,37]	3	12–43	68–88	1.14–1.39	1.10–1.25	**0.93–1.12**	2.04–2.11
Sorghum [27,33,36]	2	10–11	65–83	1.58–1.79	**0.26–0.29**	0.54–0.97	0.57–1.05
Groundnuts [27,36,37]	2	26	77–91	0.84–0.94	**0.38–0.52**	0.58–0.98	0.74–1.58
Millet [27,35,36]	1	8–17	80–90	1.70–1.73	**0.07–0.10**	0.62–1.17	0.77–1.82
WPC [27,34,36,37]	N/A	80–85	95–98	1.91–1.98	1.80–2.41	1.99–2.00	2.99–3.40
WMP [24,27,36]	N/A	28	96	1.62	1.54	1.43	1.82
